# Dicyandiamide has more inhibitory activities on nitrification than thiosulfate

**DOI:** 10.1371/journal.pone.0200598

**Published:** 2018-08-14

**Authors:** Jianfeng Ning, Shaoying Ai, Lihua Cui

**Affiliations:** 1 College of Natural Resource & Environment, South China Agricultural University, Guangzhou, China; 2 Institute of Agricultural Resources and Environment, Guangdong Academy of Agricultural Sciences, Key Laboratory of Plant Nutrition and Fertilizer in South Region, Ministry of Agriculture, Guangdong Key Laboratory of Nutrient Cycling and Farmland Conservation, Guangzhou, China; University of Delhi, INDIA

## Abstract

Dicyandiamide (DCD) and thiosulfates are two type of nitrification inhibitors (NIs) that have been widely used in agriculture to improve nitrogen (N) fertilizer use efficiency and mitigate negative effect of N on environment. Little information is available concerning the comparison of the efficacy of DCD and thiosulfate on N transformations in soil. The aim of this study was to compare the effects of DCD and thiosulfate (K_2_S_2_O_3_) on changes of NH_4_^+^-N, nitrification inhibition and N recovery in a latosolic red soil. An incubation experiment was conducted with four treatments of control (CK), N, N+DCD, and N+K_2_S_2_O_3_. Soil samples were collected periodically over 50 d to determine concentrations of mineral N, and the *amoA* gene abundance of ammonia monooxygenase (AMO) for ammonia-oxidizing bacteria (AOB) was estimated by qPCR after 10 d incubation. In the N treatment, 67.8% of the applied N as NH_4_^+^-N disappeared from the mineral N pool and only 2.7% and 30.8% of the applied N was accumulated as NO_2_^-^-N and NO_3_^-^-N, respectively. Addition of DCD and thiosulfate to the soil prevented NH_4_^+^-N disappearance by 63.0% and 13.6%, respectively. DCD suppressed the production of NO_2_^-^-N by 97.41%, whereas thiosulfate increased accumulation of NO_2_^-^-N by 14.6%. Application of N along with DCD and thiosulfate inhibited nitrification, respectively, by 72.6% and 33.1%, resulting in the delay of the nitrification process for 30 days and 10 days, respectively. Apparent N recovery in N treatment was 66.2%, which increased by 55.2% and 4.8% by DCD and thiosulfate, respectively. Numbers of AOB *amoA* gene copy was significantly inhibited by both DCD and thiosulfate, and the stronger inhibition induced by DCD than thiosulfate was recorded. Results indicated that both DCD and thiosulfate were effective inhibitors for NH_4_^+^-N oxidation, NO_3_^-^-N production, mineral N losses and AOB growth. DCD showed a more pronounced effect on nitrification inhibition than thiosulfate.

## 1 Introduction

Nitrogen is an essential element for plant growth and crop productivity in agroecosystems and undergoes a series of microbial transformations in soils. During N transformation, the nitrification plays a key role in regulating soil N loss in relation to nitrate leaching and oxynitride emissions to the environment. Conversion of ammonia to nitrite is the first and rate-limiting step in nitrification and three different groups of microorganisms including ammonia-oxidizing bacteria (AOB), ammonia-oxidizing archaea (AOA) and comammox bacteria, which all possess a pivotal enzyme-ammonia monooxy-genase (AMO) enzyme, conduct this pathway[1]. Oxidation of ammonia is considered as the main contributor to the ammonium: nitrate balance in terrestrial ecosystems, receiving much attention in difficulties relating to the chemical reactive nature of NO_2_^-^-N. Isobe et al. (2012) [2] observed the simultaneous production and consumption of NO_2_^-^, which exhibited a faster conversion rate than NH_4_^+^ and NO_3_^-^, suggesting that rapid NO_2_^-^ turnover could be a major driving force for N transformation in forest soil. It is generally accepted that NO_2_^-^ rarely accumulates in terrestrial ecosystems. However, NO_2_^-^ accumulation may occur as the consumption rate is lower than the production rate.

Nitrification inhibitors have been proved as an effective tool to reduce N loss and improve N use efficiency in last decades [3,4]. According to their modes of action on nitrification, NIs are divided into two groups: one group of chemicals inhibit the oxidation of NH_3_ to NO_2_^-^, and another group of chemicals suppress the conversion of NO_2_^-^ to NO_3_^-^. Among the chemicals available, DCD and thiosulfate are two different types of NIs, which retard the first and the second stage of nitrification, respectively. Previous work suggest that net nitrification rate was significantly related to the abundance of AOB not AOA [5], and DCD exerted more greater inhibition on the growth of AOB than AOA [6]. It was observed that the AOB rather than AOA functionally dominate NH_3_ oxidation irrespective of N-rich grassland soil [7] or high N input vegetable soil [8]. AOB is also found to be inactive in acidic conditions [9]. Thiosulfate inhibits NH_3_ oxidation by heterotrophic nitrifiers [10], however, very limited information is available on the distinct effects of thiosulfate on the growth of AOB or AOA in soil.

DCD has been evaluated as an effective NIs being widely used and studied because it is nonvolatile, nonhygroscopic, relatively water soluble and highly mobile [11]. Application of DCD increased soil N immobilization [12], urea-N recovery [13] and showed no effect on urea hydrolysis [14]. It was found that DCD was investigated mainly in the cultivated land and grassland, where applications of DCD (10–30 kg ha^-1^) have been proved to be effective in reducing N_2_O emissions from nitrogen fertilizers, urine or livestock slurry [15–19], retaining N in soil in the less mobile ammonium (NH_4_^+^) form then decreasing soil NO_3_^-^ leaching [20–22] and increasing yields [23–25]. In contrast, a few field studies showed that DCD was ineffective in mitigation on bovine urine N_2_O emission under Oxisol [26] and yield improvement of summer barley, maize, winter wheat [27], potato [28] and canola [29]. The contrastive results indicated that the performance of DCD as NIs is not consistent and is affected by many factors. According to McGeough et al. (2016) [30], among nine contrasting UK soils tested, the lower efficacy of DCD on inhibition of NH_4_^+^ oxidation and NO_3_^-^ production was observed in soils with high temperature, clay and organic matter content. In an analysis consisting 111 datasets from 39 studies, DCD was found to be effective with all fertilizer types (organic and chemical) except for nitrate-based fertilizers in different soils with irrespective of texture [31].

Thiosulfate has been identified as an active NIs, which is convenient for handling and is highly compatible with other nutrient sources, commonly used as a source of sulfur in fluid fertilizers [32]. A field study with maize showed that addition of ammonium thiosulfate to urea ammonium nitrate (UAN) tended to increase grain yield and plant nitrogen efficiency [33]. Potassium thiosulfate with application rate of 102 mg S kg^−1^ reduced N_2_O emissions by 48% indicating it is as effective as nitrification inhibitor N-Serve [34]. Ammonium thiosulfate exhibited different efficacy as incorporated with NH_4_NO_3_, (NH_4_)_2_SO_4_, urea and poultry manure, and the highest inhibitory effect was with NH_4_NO_3_ which maintained 100% inhibition of nitrification during 12 weeks [35]. Inhibition of nitrification induced by sodium thiosulfate ranged 55–80% with application of 32 mg S kg^-1^ [36]. During nitrification inhibition, thiosulfate resulted in the accumulation of NO_2_^-^ and NO under aerobic conditions and showed no effect on reduction rate of NO_3_^-^ under anaerobic environment [37]. In addition, thiosulfate was found to retard soil urease activity when applied at rates of 2500 or 5000 μg g^-1^ soil [38]. As a nitrification inhibitor, thiosulfate acts more efficiently than *Azadirachta indica* (neem) and calcium chloride [39], but not as DCD. The stronger inhibitory effect induced by DCD than thiosulfate was observed by Goos and Johnson (1992) [40] under laboratory, field microplot, and field conditions. Kumar et al. (2000) [41], however, found that DCD and thiosulfate reduced N2O emissions by a similar amount from urea (11 and 9% reduction, respectively) in a rice field. The different effects of DCD and thiosulfate on nitrification attribute to not only their distinct characteristics but also the various modes of action [42,43]. Moreover, the efficacy of NIs can vary widely with the variation of environmental factors, while the literature relating to DCD and thiosulfate is extensive, there have been few studies comparing their ability on N transformations involving NO_2_^-^-N dynamics and ammonia-oxidizing microbes under same environmental conditions, and the mechanism for the differentiation between the efficacy of DCD and DMPP is still unclear.

In this study, both DCD and thiosulfate (K_2_S_2_O_3_) were chosen because of their importance as NIs in agriculture and horticulture. We hypothesized that DCD and thiosulfate may impose different effects on N transformation based on their different mechanisms related to nitrification inhibition. Few studies have been examined the impact of thiosulfate on the abundance of ammonia-oxidizing microbes, which limits our comprehensive understanding of its potential in nitrification inhibition and N management. The abundance of AOB in different treatments was investigated because of its dominance during ammonia oxidation in vegetable soils as reported before [8]. The objectives of this study were to: i) test if thiosulfate is as effective as DCD acting as a nitrification inhibitor, ii) assess the different effect between DCD and thiosulfate on NO_2_^-^-N accumulation, NO_3_^-^-N production and the abundance of bacteria (AOB), iii) ascertain whether thiosulfate imposes inhibitory effect on ammonium oxidation.

## 2 Materials and methods

### 2.1 Soil and nitrification inhibitors

Latosolic red soil used in this incubation experiment was collected from an experimental vegetable farm (23°08tos^″8^N, 113°20 11o E) at the Institute of Agricultural Resources and Environment, Guangdong Academy of Agricultural Sciences, Guangzhou City, China. The farm grows mainly leafy vegetables such as Chinese flowering cabbage, romaine lettuce, spinage, which has been cultivated for more than 7 years. This soil is loamy clay (clay 27.8%, silt 24.1%, sand 48.1%) with pH of 5.73, 17.07 mg g^-1^ organic C, 1.56 mg g^-1^ total N, 37.0 ug g^-1^ NH_4_^+^-N, 0.35 ug g^-1^ NO_2_^-^-N and 56.0 ug g^-1^ NO_3_^-^-N. The field-moist soil was air-dried and sieved (< 2 mm) after removing the stones and plant materials.

The nitrification inhibitor used was DCD (J&K, 99.5%) and potassium thiosulfates (Sigma-Aldrich, >95%): K_2_S_2_O_3_.

### 2.2 Experimental design and soil incubation

One kilogram of air-dried soil was moistened to 40% water-holding capacity with deionized water and pre-incubated at 20°C for 2 weeks to stabilize the microbial activity. The experiment consisted of six N treatments, namely no amendment (CK), N, N+DCD, and N+K_2_S_2_O_3_. Eight incubation periods, i.e., 1, 5, 10, 15, 20, 30, 40 and 50 d were also used in this study and each treatment was replicated three times. A total of 96 experimental units were prepared at the beginning of the experiment. After pre-incubation, soil sample of 50 g (on a dry weight basis) was weighed and placed into plastic jar (250 ml). Urea (46% N) was supplied as the N source to all jars except CK at a rate of 200 mg N kg ^-1^ soil. The application rate of DCD and thiosulfate (K_2_S_2_O_3_) was 40 mg kg^-1^ (20% of N applied), and 64 mg S kg^-1^ soil [35], respectively. All the chemicals were dissolved in deionized water and then mixed well with the soil. The soil was adjusted to 60% water-holding capacity. All of the jars were covered by parafilm with 4 small holes on the top for aeration and incubated at 20°C under dark conditions. During incubation period, soil moisture was maintained at 60% by replenishing the required water every 2 days.

### 2.3 Soil analysis

#### 2.3.1 Mineral N analysis

Concentrations of mineral N, including NH_4_^+^-N, NO_2_^-^-N and NO_3_^-^-N, were analyzed. The mineral N was extracted with 2 M KCl solution (10:1, KCl: fresh soil). The NH_4_^+^-N and NO_3_^-^-N were determined with a flow-injection autoanalyzer (Alliance-Futura II, France). The NO_2_^-^-N content in the extracts was assayed colorimetrically by the Griess-Ilosvay methods. Soil water content was determined after drying at 105°C for 48 h.

#### 2.3.2 DNA extraction and quantitative PCR

After 10 d incubation, three replicate jars of each treatment were selected to extract DNA and analysis of *amoA* genes was performed by Real-time, quantitative PCR (SYBRGreen-based qPCR). The total DNA was extracted from 0.5 g of soil by using the FastDNA™ Spin Kit for Soil (MP Biomedicals, United States), according to the manufacturer’s protocol. The quality and the purity of DNA were verified with a spectrophotometer (NanoDrop2000, Thermo Fisher Scientific, United States). Quantitative PCR of *amoA* genes was performed to estimate the abundance of the ammonia-oxidizing bacterial community. The primers *amoA*-1F (51F s was performed to e) and *amoA*-2R (5R CCCCTCKGSAAAGCCTTCTTC-3e) were used for ammonia-oxidizing bacteria generating a 491 bp fragment were used for generating a 635 bp fragment [44]. The abundance of *amoA* genes of AOB was determined by qPCR (ABI7500, Applied Biosystems, United States). Each 20 μL qPCR reaction contained 16.4 μL 2×ChamQ SYBR Color qPCR Master Mix (Vazyme, Nanjing, China), 0.8 μL of the specific forward and reverse primer (5 μM), 2 μL of template DNA. The fragments for AOB was amplified with an initial denaturation step at 95°C for 5 min, followed by 40 cycles of 5 s at 95°C, 30 s at 58°C, 40 s at 72°C for the collection of fluorescence data. The standard curves for the AOB was obtained using serial dilutions of 10-fold serial dilutions of a known copy numbers of the plasmid DNA. The PCR reaction runs had an efficiency of 97.04%, and the R^2^ value was 0.9988.

### 2.4 Calculations

Percentage of NH_4_^+^-N disappearance from mineral N pool and the proportion of NH_4_^+^-N converted to NO_2_^-^-N or NO_3_^-^-N were calculated according to the method of Abbes et al. (1994) [45] with some modifications as below:

$$\%NH_{4}^{+}-N_{disappearance}=100-100\times \frac{\left(NH_{4}^{+}-N_{treat}\right)-\left(NH_{4}^{+}-N_{ck}\right)}{\mathrm{Nu}}$$ (1)

$$\%NH_{4}^{+}-N\;\mathrm{conversion}\;to\;NO_{2}^{-}-N=100\times \frac{\left(N0_{2}^{-}-N_{treat}\right)-\left(N0_{2}^{-}-N_{ck}\right)}{\mathrm{Nu}}$$ (2)

$$\%NH_{4}^{+}-N\;\mathrm{conversion}\;to\;NO_{3}^{-}-N=100\times \frac{\left(N0_{3}^{-}-N_{treat}\right)-\left(N0_{3}^{-}-N_{ck}\right)}{\mathrm{Nu}}$$ (3)

where N_u_ is the added urea-N (200 mg kg^-1^), N_treat_ is the N in the treatments and N_ck_ is the N in the control.

Percentage of the apparent nitrification rate was calculated with the following Eq.:

$$Apparent\;nitrification\;rate\;(\%)=100\times \frac{NO_{3}^{-}-N}{\left(NH_{4}^{+}-N+NO_{3}^{\begin{array}{c} -\\ - \end{array}}-N+NO_{2}^{-}-N\right)}$$ (4)

Percentage of nitrification inhibition rate was calculated based on the method of McCarty and Bremner (1989) [46] with some modifications:

$$\mathrm{Nitrification}\;\mathrm{inhibition}\;\mathrm{rate}\;\left(\%\right)=100\times \frac{\left(C-T\right)}{C}$$ (5)

where *C* is the amount of NO_3_^-^-N produced in soil amended with urea-N, *T* is the amount of NO_3_^-^-N produced in soil amended with urea-N and NIs.

### 2.5 Data statistics

All data were the means of three replications, which were expressed as mean ± standard error. ANOVA was performed using SAS software (1999) and significant differences among means were determined by Fisher’s least-significant difference test (LSD) at *P* ≤ 0.05.

## 3 Results and discussion

### 3.1 Reduction of NH_4_^+^-N

**Fig 1:**
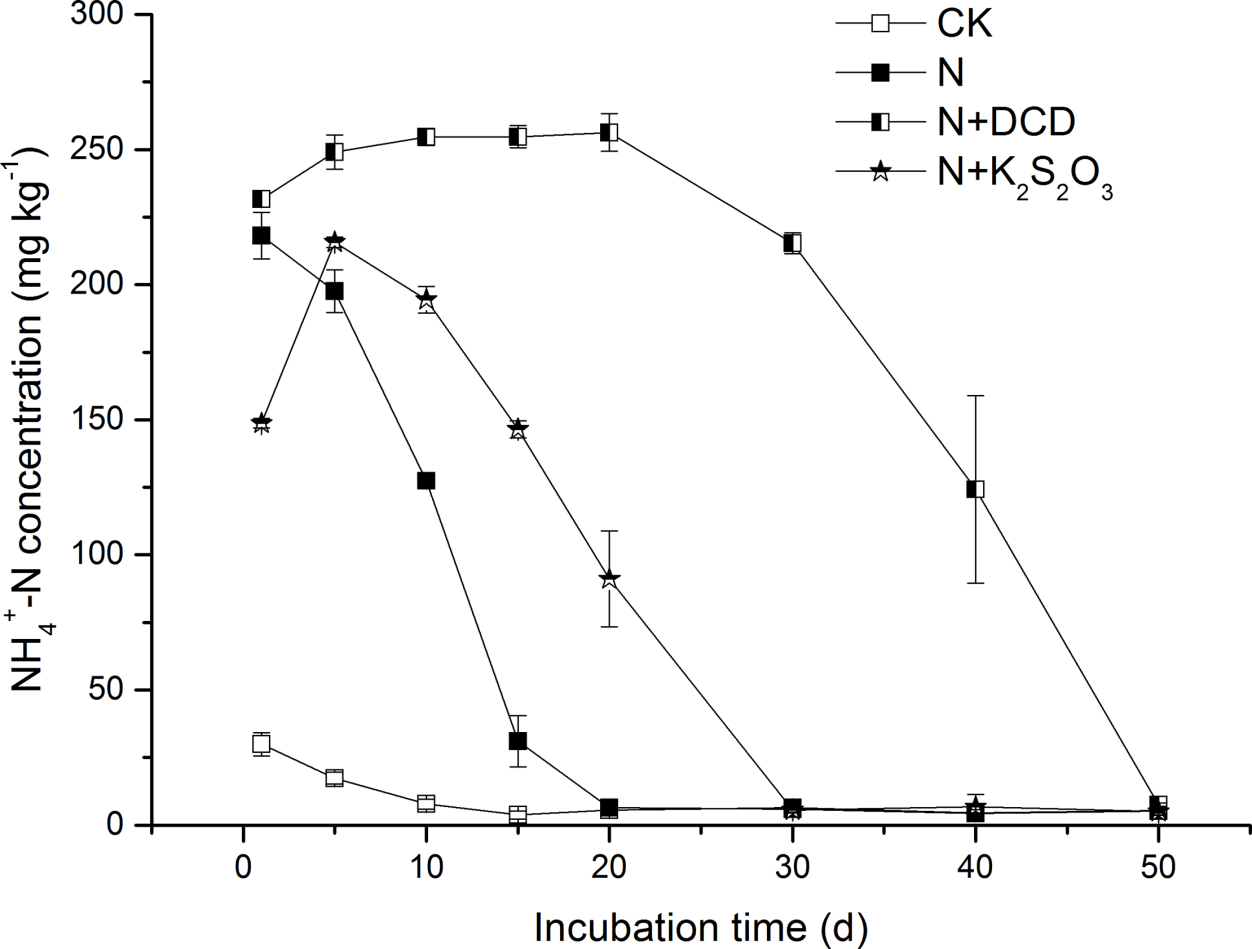
Dynamic changes in the concentration of NH_4_^+^-N in soil amended with urea N with or without nitrification inhibitor. Soil added with N with or without nitrification inhibitor (DCD, K_2_S_2_O_3_) were incubated under dark conditions of 20°C (see details in Materials and Methods**)**. Three soils samples in each treatment were taken after 1, 5, 10, 15, 20, 30, 40 and 50 days of incubation. Data in the figure represent means ± SD (n = 3).

The initial concentration of soil NH_4_^+^-N (29.9 mg kg^-1^) in the CK treatment decreased quickly with time and reached 5.4 mg kg^-1^ at the end of the experiment (Fig 1). It was indicated that NH_4_^+^-N in the CK soil decreased by 81.94% due to the nitrification as observed from the increment of NO_3_^-^-N. Application of urea significantly increased initial NH_4_^+^-N concentration in soil amended by N with or without NIs. The highest concentration of NH_4_^+^-N in the N treatment was 218.1 mg kg^-1^ at the beginning of the experiment (Day 1) and decreased to the initial CK level at day 20 (Fig 1). It was indicated that urea was hydrolyzed within 24 hours. A similar hydrolysis rate of urea was also observed in the study of Gould et al. (1973) [47], where urea was hydrolyzed within 20 hours in several Alberta soils when applied as a solution. In contrast, the concentration of NH_4_^+^-N in the treatment of N+K_2_S_2_O_3_ increased from 148.8 mg kg^-1^ at Day 1 to the maximum level of 215.7mg kg^-1^ at Day 5, suggesting that thiosulfate application resulted in 4-day delay of urea hydrolysis as compared to the N treatment (Fig 1). This was in agreement with the results reported by Goos (1985) [36] where urea hydrolysis was inhibited by thiosulfate during the 2–4 d incubation period. Results from this study also confirmed that thiosulfate is a urease inhibitor. During the whole incubation period, the NH_4_^+^-N concentration in the N+DCD treatment was higher than that in other treatments (Fig 1). Particularly, NH_4_^+^-N concentration increased progressively from 231.7 mg kg^-1^ at Day 1 to 256.3 mg kg^-1^ at Day 20, which could be attributed to the NH_4_^+^-N production derived from the decomposition of DCD [11], as urea hydrolysis has been proved unaffected by DCD [14].

**Fig 2:**
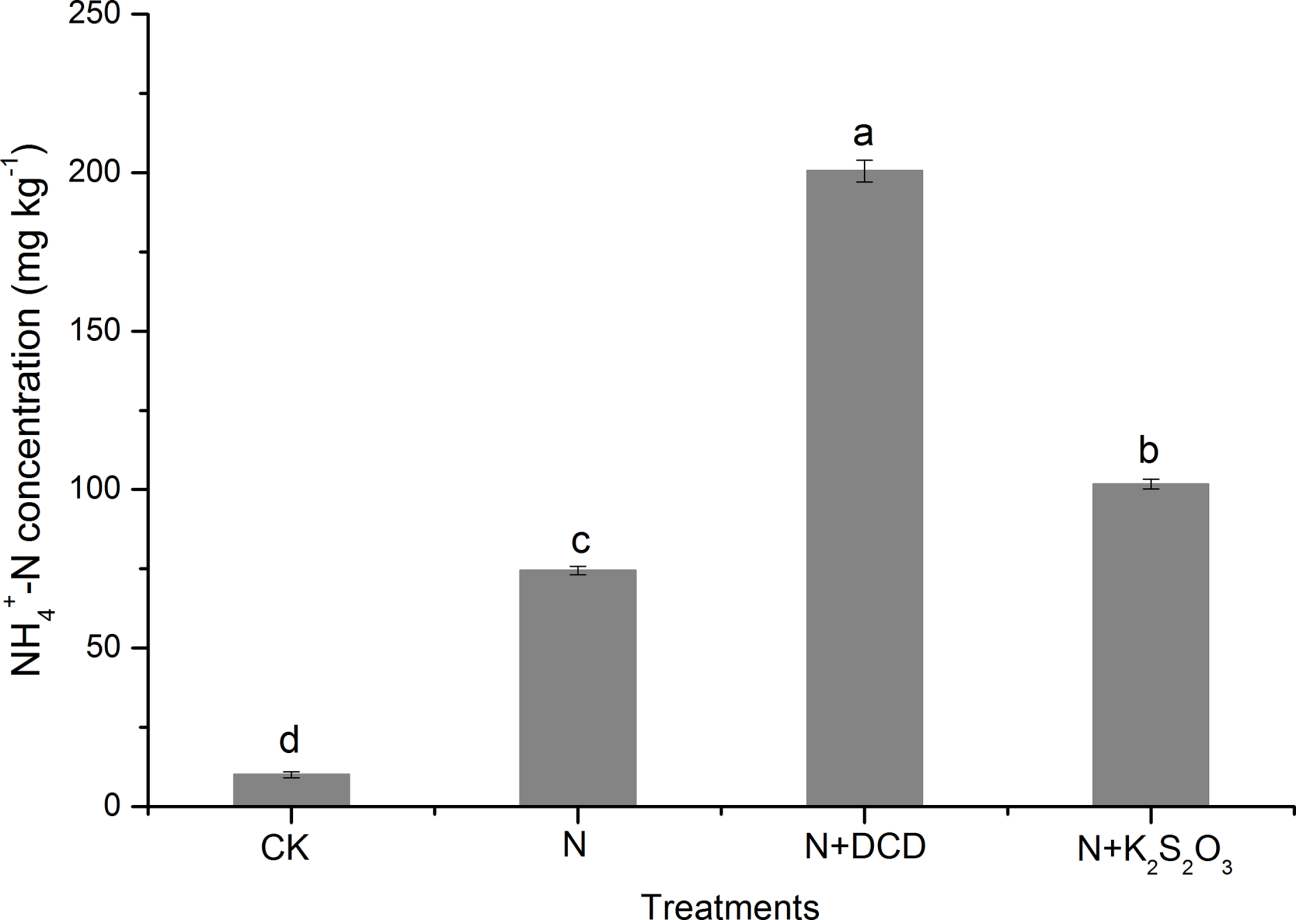
Overall changes (B, average over 50 d incubation) in the concentration of NH_4_^+^-N in soil amended with urea N with or without nitrification inhibitor. Data in the figure represent means ± SD (n = 3). Different letters on the column indicate statistical difference according to Fisher^’^s least-significant difference test (*P*≤0.05).

Averaged across different timings, the NH_4_^+^-N concentration in CK soil was 10.1 mg kg^-1^ (Fig 2), which significantly increased to 74.5, 200.5, and 101.7 mg kg^-1^ in soil of N, N+DCD, and N+K_2_S_2_O_3_, respectively. As the NH_4_^+^-N concentration in the CK treatment was deducted, the NH_4_^+^-N in N treatment was 64.4 mg kg^-1^ indicating that 67.8% of the applied N (200 mg kg^-1^) had disappeared from the mineral N pool. Addition of DCD and thiosulfate decreased NH_4_^+^-N disappearance by 63.0% and 13.6% as compared to that of N treatment. The decrease in NH_4_^+^-N loss was mainly due to the nitrification inhibition induced by NIs then maintained NH_4_^+^-N for longer time in the mineral N pool. DCD was more effective than thiosulfate in preventing NH_4_^+^-N disappearance from the mineral N pool.

### 3.2 Accumulation of NO_2_^-^-N

**Fig 3:**
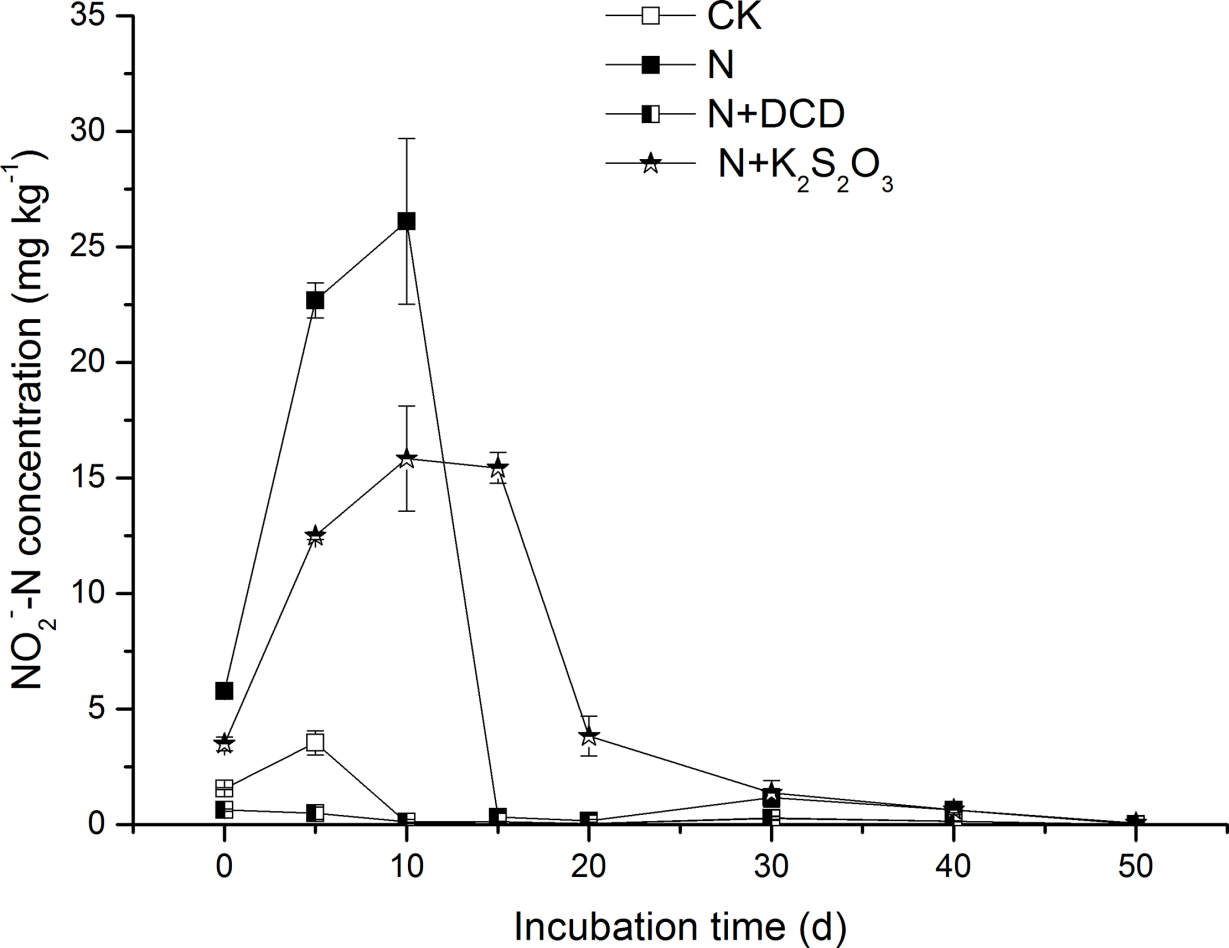
Dynamic changes in the concentration of NO_2_^-^-N in soil amended with urea N with or without nitrification inhibitor. Soil added with N with or without nitrification inhibitor (DCD, K_2_S_2_O_3_) were incubated under dark conditions of 20°C (see details in Materials and Methods**)**. Three soils samples in each treatment were taken after 1, 5, 10, 15, 20, 30, 40 and 50 days of incubation. Data in the figure represent means ± SD (n = 3).

During the whole incubation period, a considerable proportion of NH_4_^+^-N loss from the mineral N pool which accompanied with the production of NO_2_^-^-N (Fig 3). It was observed that a large NO_2_^-^-N accumulation with nitrification of urea-N normally occurred, which could be associated with high pH and NH_4_^+^ concentrations during urea hydrolysis leading to the inhibition of NO_2_^-^ oxidizers [48]. In this study, urea was used as the N source and the accumulation of NO_2_^-^-N in the N treatment occurred mainly in the early stage of incubation and the peak concentration of 26.1 mg kg^-1^ was recorded at Day 10. The maximum NO_2_^-^-N concentration was 13.1% of the applied urea-N, which was higher than that in the soil of Waurn Ponds c (II) reported by Magalhães et al. (1987)[49]. NIs have been proved to be an important factor in regulating NO_2_^-^ content in soils [50]. Thiosulfate retards the conversion of NO_2_^-^-N to NO_3_^-^-N, resulting in a substantial amount of NO_2_^-^-N in the soil (Fig 3). However, Shen et al. (2003) [48] found that NO_2_^-^ occurrence in different soils was suppressed significantly with application of nitrapyrin or sodium azide. Similarly, no peak concentration of NO_2_^-^-N was observed in the N+DCD treatment (Fig 3), confirming that DCD inhibited the NO_2_^-^-N production by directly suppressing the activity of ammonia oxidizing enzymes and indirectly repressing the growth of ammonia oxidizers [51].

**Fig 4:**
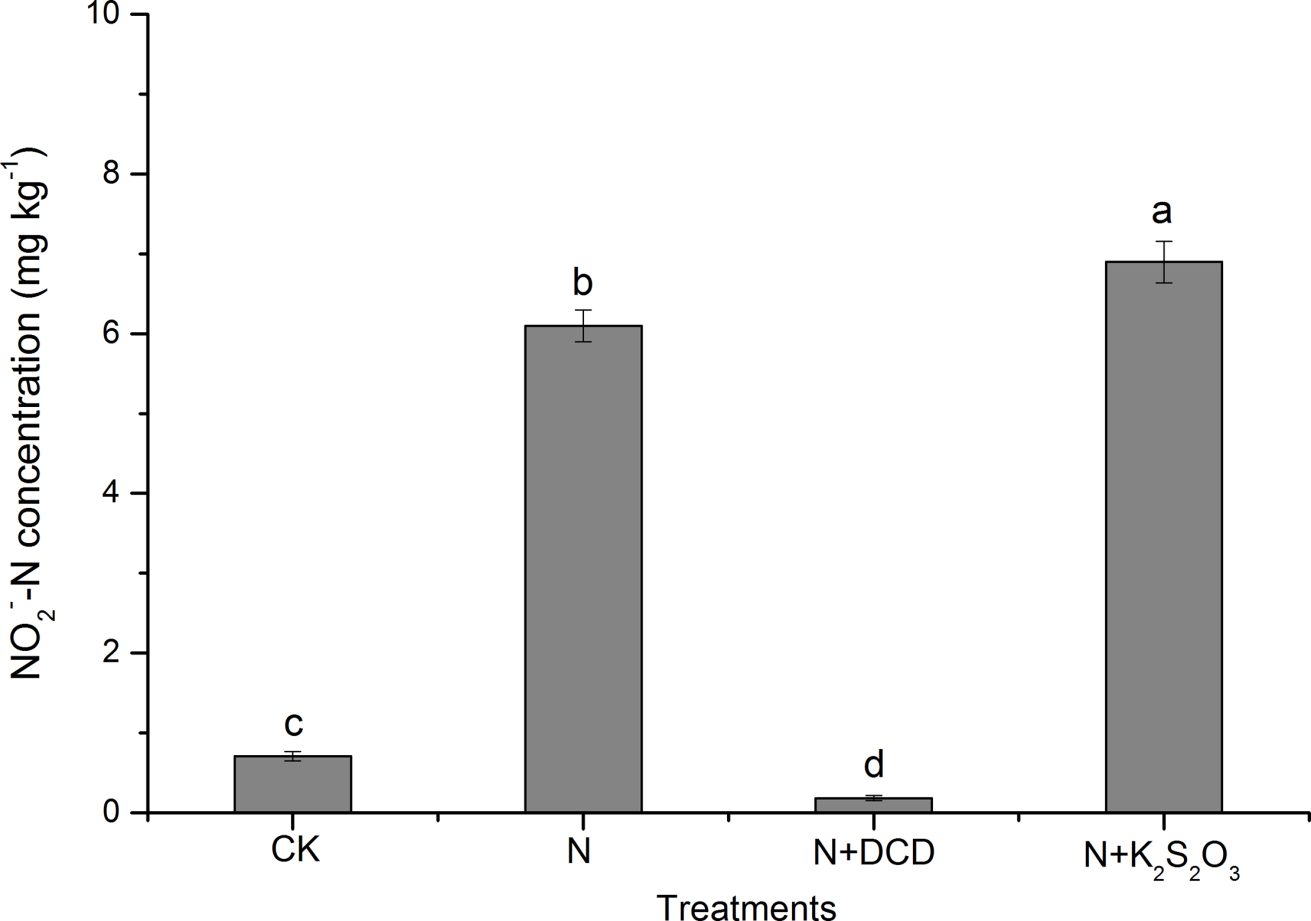
Overall changes in the concentration of NO_2_^-^-N in soil amended with urea N with or without nitrification inhibitor. Data in the figure represent means ± SD (n = 3). Different letters on the column indicate statistical difference according to Fisher^’^s least-significant difference test (*P*≤0.05).

Overall treatment effects showed that NO_2_^-^-N in soil of CK was 0.71 mg kg^-1^ (Fig 4), which was in accordance with Magalhães et al. (1987) [49] who found the NO_2_^-^-N concentration was less than 1 mg kg^-1^ in unfertilized soils. Considering NO_2_^-^-N in the CK soil, NO_2_^-^-N in N, N+DCD and N+K_2_S_2_O_3_ was 5.4, -0.53, and 6.19 mg kg^-1^, respectively. Apparently, the results indicated that in N treatment 2.7% of applied N (200 mg kg^-1^) was accumulated as NO_2_^-^-N, which was increased by 14.6% in soil of N+thiosulfate.

### 3.3 Production of NO_3_^-^-N and nitrification inhibition

**Fig 5:**
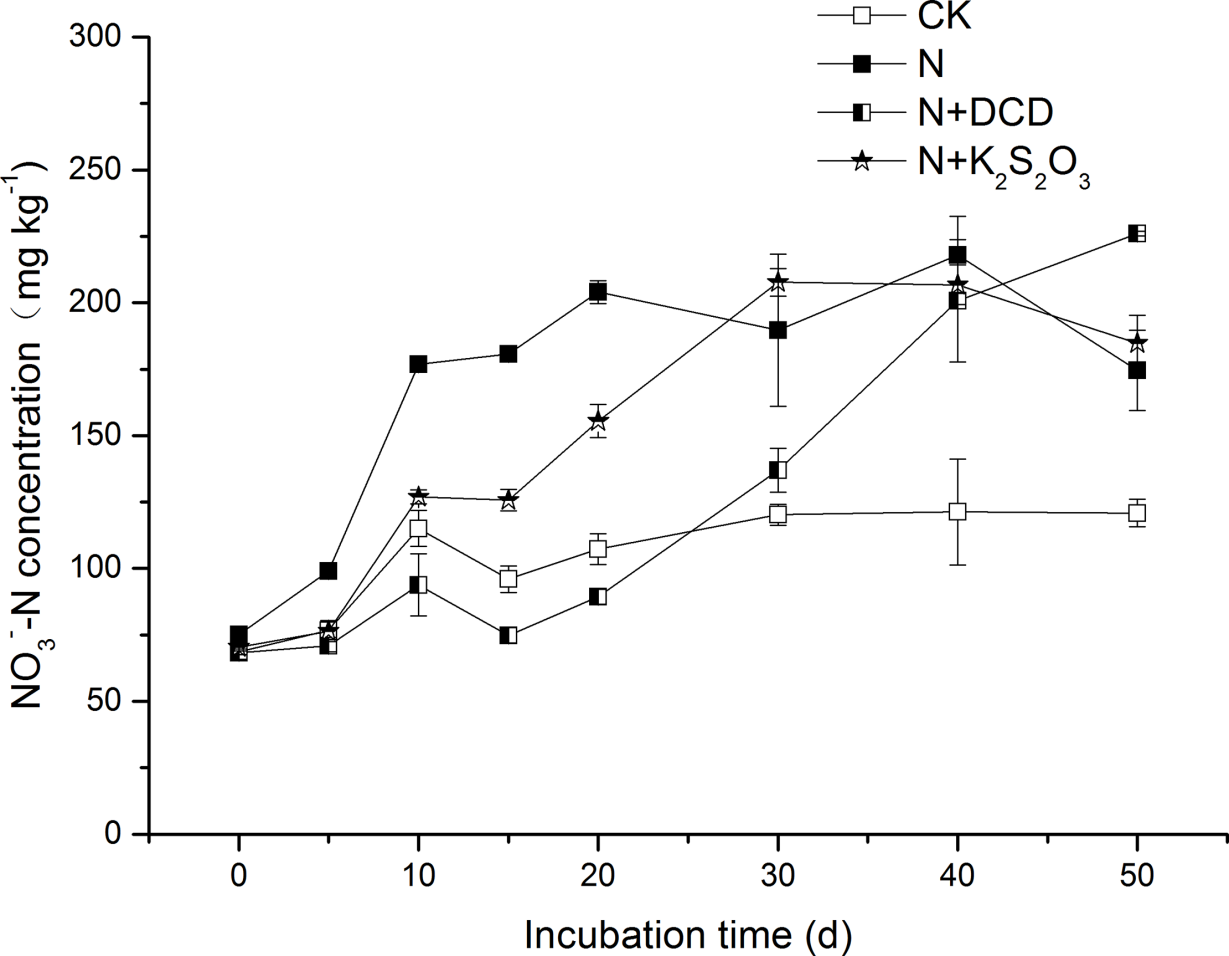
Dynamic changes in the concentration of NO_3_^-^-N in soil amended with urea N with or without nitrification inhibitor. Soil added with N with or without nitrification inhibitor (DCD, K_2_S_2_O_3_) were incubated under dark conditions of 20°C (see details in Materials and Methods**)**. Three soils samples in each treatment were taken after 1, 5, 10, 15, 20, 30, 40 and 50 days of incubation. Data in the figure represent means ± SD (n = 3).

**Fig 6:**
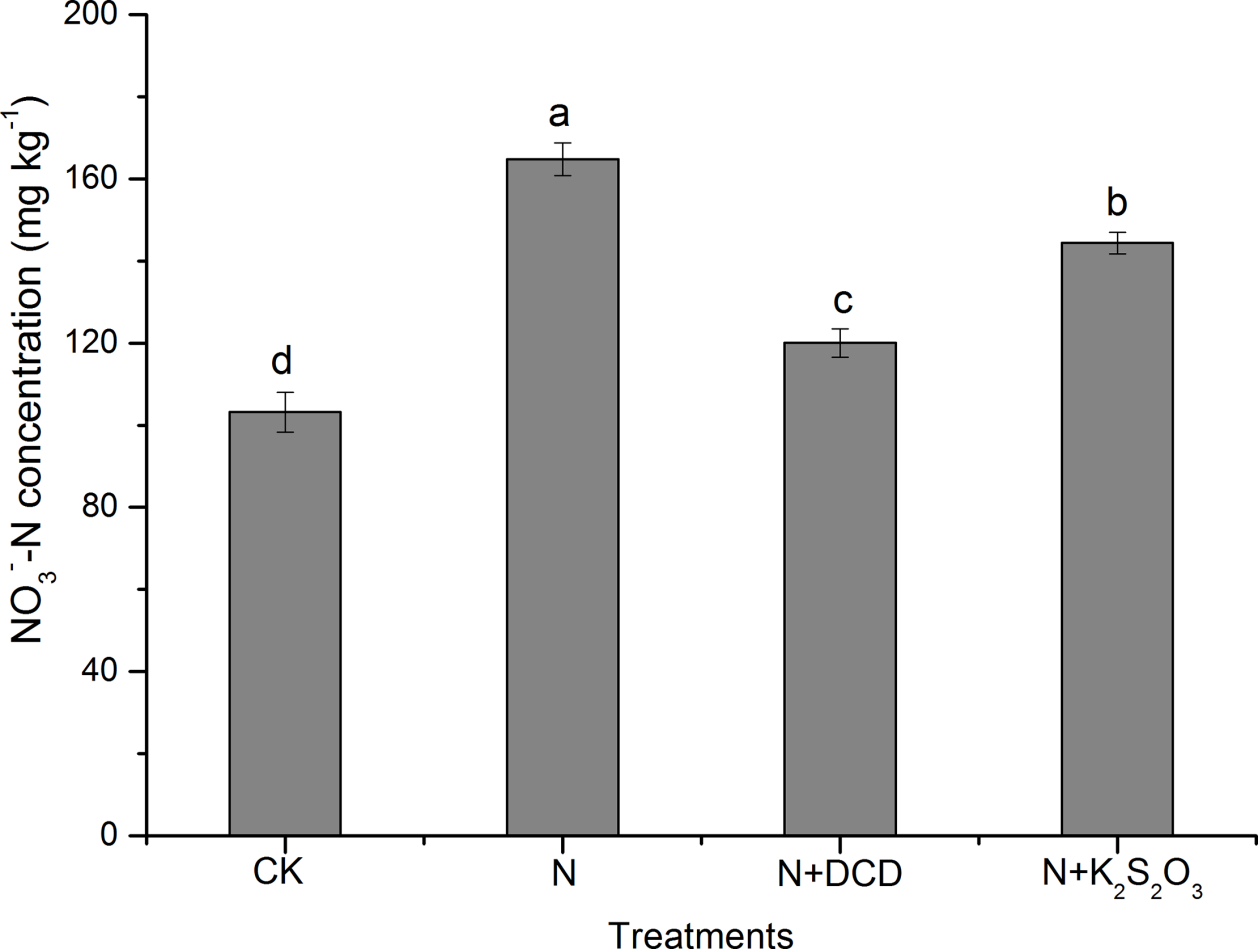
Overall changes in the concentration of NO_3_^-^-N in soil amended with urea N with or without nitrification inhibitor. Data in the figure represent means ± SD (n = 3). Different letters on the column indicate statistical difference according to Fisher^’^s least-significant difference test (*P*≤0.05).

Along with the nitrification process, accumulation of NO_3_^-^-N in the soil for different treatments increased progressively (Fig 5). In the CK treatment, the maximum NO_3_^-^-N of 120.8 mg kg^-1^ was observed at the end of the experiment, implying a 76.1% increase as compared to that of the initial stage (Fig 5). A higher NO_3_^-^-N concentration within 20 days in the N treatment relative to that of other treatments was observed. Particularly, the apparent nitrification rate of 96.9% in the N treatment reached to that of the CK level at Day 20 (Table 1), indicated that nitrification process was completed within 20 days. Similarly, the nitrification process in N+K_2_S_2_O_3_ and N+DCD was completed within 30 days and 50 days, suggested that thiosulfate and DCD delayed N nitrification process for 10 days and 30 days, respectively. Based on average across different timing estimates, accumulation of NO_3_^-^-N in the CK treatment was 103.2 mg kg^-1^, and a significant increase of NO_3_^-^-N content in the soils for the N and N+NIs treatments was observed (Fig 6). Taking into account the NO_3_^-^-N concentration of 103.23 mg kg^-1^ in the CK treatment, the NO_3_^-^-N production in the N treatment was 61.5 mg kg^-1^, indicating that 30.8% of applied N (200 mg kg^-1^) was transformed to NO_3_^-^-N. It is worth noting that NO_3_^-^-N accumulation was much lower than the NH_4_^+^-N depletion (67.8% of added N). This is in a good agreement with the study of Abbasi and Adams (2000) [52] who found that more than 58% of N applied as NH_4_^+^-N was depleted within 50 days and only 21% of NH_4_^+^-N was accumulated as NO_3_^-^-N. As the NO_3_^-^-N in the CK treatment soil was deducted, concentration of NO_3_^-^-N in the soil of N+DCD and N+K_2_S_2_O_3_ treatments was 16.9, and 41.2 mg kg^-1^ (Fig 6), respectively. It was indicated that DCD and K_2_S_2_O_3_ inhibited nitrification by 72.6 and 33.1%, respectively.

**Table 1:** Apparent nitrification rate of urea N applied to soil with or without nitrification inhibitor.

Treatment ^a^	Incubation time (d)							
	1	5	10	15	20	30	40	50
**CK**	68.61 a	78.62 a	93.59 a	96.16 a	94.97 a	94.73 b	96.19 a	95.73 b
**N**	25.13 d	31.03 b	53.56 b	85.35 b	96.91 a	96.23 ab	97.79 a	97.05 a
**N+DCD**	22.70 d	21.20 d	26.87 d	22.69 d	25.85 d	38.84 c	62.01 b	96.82 ab
**N+K**_**2**_**S**_**2**_**O**_**3**_	31.62 bc	25.08 c	37.65 c	43.70 c	62.29 bc	96.87 a	96.63 a	97.35 a

^a^ Data in the table represent means of three replicates (n = 3).

Data followed by different letters in the same column are statistically different according to Fisher’s least-significant difference test (*P*≤0.05).

Results from this study implied that DCD exerted more pronounced effect on nitrification inhibition than that of thiosulfate. It is known that the efficacy of NIs normally depend on their persistence in the soil. In the previous studies, DCD or thiosulfate was found to be degraded predominately by microbes in soils [53,54]) where temperature is a major factor. The half-life period of DCD was 100 days under 10°C [55] and decreased to 30–40 days under 20–30°C conditions [56]. However, the persistence of thiosulfate was 25 days in soil with 25°C [37]. According to Chaves et al. (2006) [11], the inhibitory effect induced by DCD can be sustained up to 50 days under optimal conditions. In the present study, the stronger capability of nitrification inhibition in DCD than thiosulfate could be partly attributed to the difference of their persistence in the soil. Increase of nitrification rate with time (Table 1) indicated indirectly the degradation of DCD or thiosulfate in the soil.

### 3.4 Change of total mineral N

**Fig 7:**
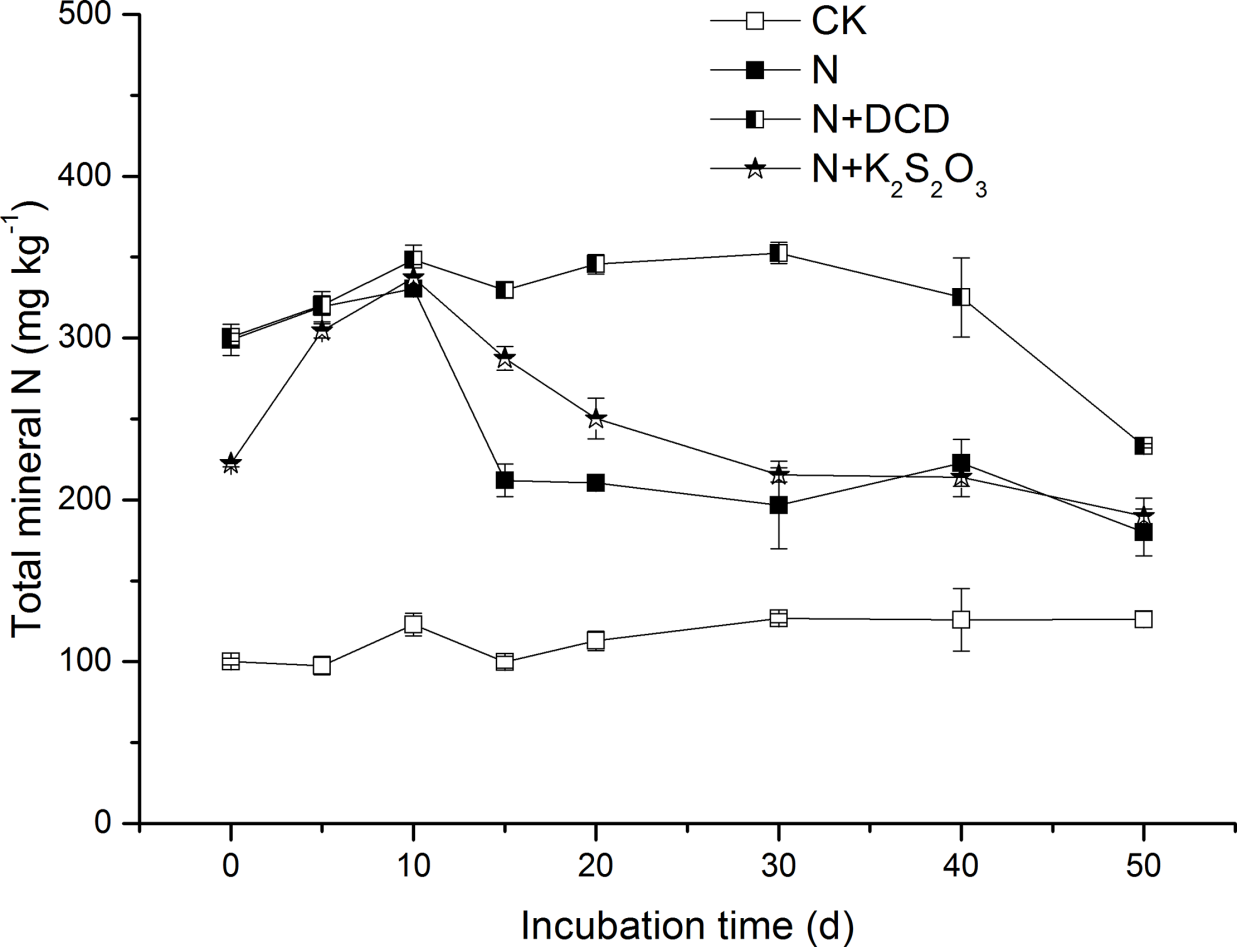
Dynamic changes in the concentration of total mineral nitrogen in soil amended with urea N with or without nitrification inhibitor. Soil added with N with or without nitrification inhibitor (DCD, K_2_S_2_O_3_) were incubated under dark conditions of 20°C (see details in **Materials and Methods**). Three soils samples in each treatment were taken after 1, 5, 10, 15, 20, 30, 40 and 50 days of incubation. Data in the figure represent means ± SD (n = 3).

In the CK treatment soil, the initial total mineral N (TMN) was 100.1 mg kg^-1^ and increased slowly as time elapsed, and finally reached a maximum concentration of 126.9 mg kg^-1^ at Day 30, indicating a 26.8% increase compared to that at the initial TMN concentration (Fig 7). Application of N significantly increased the soil initial TMN concentration. The peak concentrations of 330.4 and 337.27 mg kg^-1^ were observed at Day 10 for the treatments of N and N+K_2_S_2_O_3_, respectively (Fig 7). The TMN in the N+DCD treatment showed different changing patterns as its maximum value of 352.6 mg kg^-1^ was recorded at day 30. Average across different timings, the TMN in the N treatment was 246.4 mg kg^-1^ (Table 2). In consideration of the mineral N in the CK soil (114.0 mg kg^-1^), TMN in the N treatment was 132.3 mg kg^-1^, suggesting that 66.2% of the applied N (200 mg kg^-1^) had been recovered from the mineral N. Application of N with nitrification inhibitors increased N recovery. As compared to the N treatment, soil N recovery rate in the soils for the N+DCD and N+K_2_S_2_O_3_ treatments was increased by 55.2% and 4.8% (Table 2), respectively. Particularly, the TMN in the N+DCD treatment exceeded 200 mg kg^-1^ with a N recovery rate of 102.7%, indicating the replenishment of total mineral N pool. In a previous report, Vilsmeier et al. (1987) [56] found that N recovery was increased by 23%, 30% and 61% in a soil amended with nitrification inhibitor phenylacetylene, nitrapyrin and 2-ethynylpyridine. A 45% increase in the recovery of fertilizer N with application of coated calcium carbide has been reported by Freney et al. (1992) [57]. These authors found that DCD was as efficient as 2-ethynylpyridine and coated calcium carbide, and was superior over thiosulfate in improving N recovery. An increase of N recovery resulted in the decrease of N which was mainly attributed to the ability of nitrification inhibitor to inhibit the production of NO_3_^-^-N and then restricted the supply of NO_3_^-^-N to denitrifying organisms, leading to a reduction of N loss through denitrification. The similar results were also found by Abbasi et al. (2011) [39] and Cahalan et al. (2015) [58], where soil N_2_O emissions were reduced significantly by NIs.

**Table 2:** Apparent recovery of fertilizer N applied to soil with or without nitrification inhibitor.

Item^b^	CK	N	N+DCD	N+K_2_S_2_O_3_
**TMN** **(mg kg**^**-1**^**)**	114.04 d	246.36 c	319.46 a	252.74 bc
**TMNR** **(mg kg**^**-1**^**)**	-	132.32 c	205.42 a	138.7 bc
**ARUN (%)**	-	66.16 c	102.71 a	69.35 bc

^b^ Data in the table represent means of three replicates (n = 3).

Data followed by different letters in the same line are statistically different according to Fisher’s least-significant difference test (*P*≤0.05). TMN, Total mineral nitrogen (NH_4_^+^-N+NO_2_^-^-N+NO_3_^-^-N, mean of 50 d); TMNR, TMN recovered from applied N; ARUN, apparent recovery of urea N.

### 3.5. Ammonia-oxidizing bacterial (AOB) *amoA* gene copies

**Fig 8:**
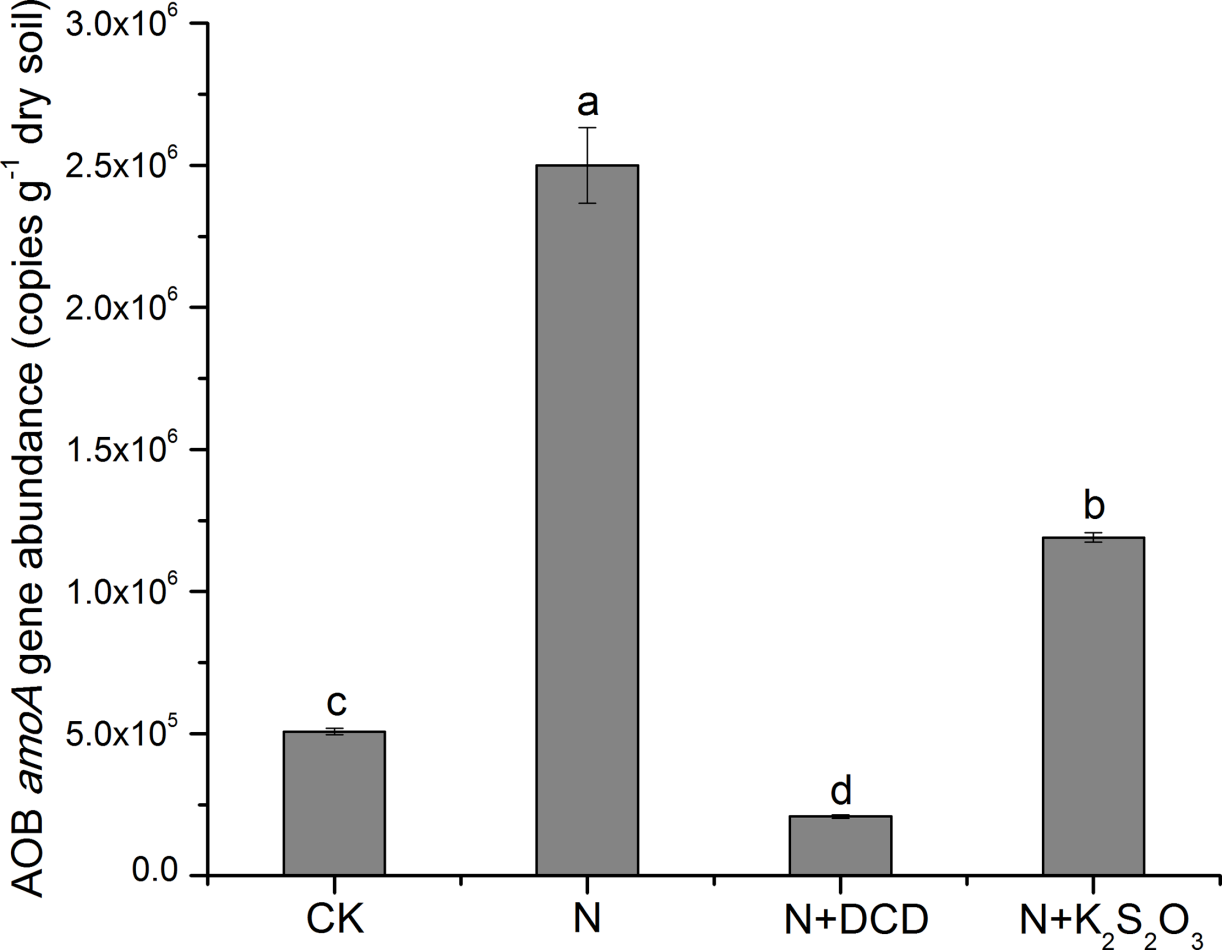
Bacterial *amoA* gene copies in soil amended with urea N with or without nitrification inhibitor. Data in the figure represent means ± SD (n = 3). Different lowercase means statistically different according to Fisher’s least-significant difference test (*P*≤0.05).

The abundance of AOB in different treatments was determined after 10-d incubation (Fig 8). AOB *amoA* gene copy number was 5.07×10^5^ copies g^−1^ dry soil in the CK treatment, which is in the range of previous reports that soil typically contains 10^4^−10^6^ bacterial ammonia oxidizers g^-1^ [59]. Application of urea resulted in significantly increase in AOB *amoA* gene copies relative to the CK, which is mainly attribute to the high dosage of ammonia (NH_3_), the substrate of AOB *amoA*, that produced from the hydrolysis of urea. The similar results were also observed by Di et al. [7,60]. The AOB growth in soil treated with urea in conjunction with NIs was restrained significantly, and the reduction of 91.69% and 52.34% in treatment of N+DCD and N+K_2_S_2_O_3_ was observed, respectively, when compared to that of N treatment (Fig 8). Particularly, AOB *amoA* gene copy number of 2.08×10^5^ copies g^−1^ dry soil in N+DCD was significantly lower than that of CK treatment. Responses of AOB abundance indicated that bacteria growth was promoted by urea application while was restrained by nitrification inhibitor DCD and K_2_S_2_O_3_, indicating that thiosulfate is capable to retard the oxidation of ammonia, although it exerted a weaker inhibitory effect than DCD. According to Amberger (1989) [61], the nitrification inhibitor DCD inhibits the ammonium oxidation via deactivating the ammonia monooxygenase (AMO) enzyme of the AOB, retarding the ammonia oxidation. Growth inhibition of AOB with DCD has been observed in a number of previous results [5,17,60,62]. As for thiosulfate, the inhibitory function is possibly associated with the direct toxic effect of itself or its oxidation products (tetrathionate or carbon disulfide) on nitrifiers, or the indirect effects of volatile organic sulfur compounds produced from transformation of thiosulfate inhibiting on NH_3_ monooxygenase activity [33,37,63]. As observed from the changes of mineral N (Figs 1–6), as well as the AOB *amoA* copy number (Fig 8) in treatment of N+DCD and N+K_2_S_2_O_3_, there is increasing evidence to suggest that thiosulfate possesses the inhibitory ability on oxidation of both ammonia and nitrite, the two steps of nitrification process. Difference between the efficacy of DCD and thiosulfate may be attribute to a various factors, i.e. their half-life period, the sorption property as well as their ability on substrate utilization in soil, and the further research is required to understand the main factors relating the efficacy of this two nitrification inhibitors.

## 4 Conclusions

The present study showed that both DCD and thiosulfate (K_2_S_2_O_3_) were effective inhibitors for the NH_4_^+^-N oxidation, NO_3_^-^-N production and mineral N losses. The mechanisms were due to the inhibition of AOB growth by DCD and thiosulfate resulting in the retardation of nitrification and thus keeping the NH_4_^+^-N for longer period in the mineral N pool. We found that thiosulfate increased accumulation of NO_2_^-^-N, while DCD inhibited production of NO_2_^-^-N. In general, DCD exhibited a more pronounced effect in regulating N transformations than K_2_S_2_O_3._ The efficacy of NIs was normally affected by soil temperature, moisture, microbial activity as well as many other environmental factors, further study should be conducted to investigate the effects of DCD and thiosulfates on growth and N use efficiency of crops under field conditions.

## Supporting information

S1 Table Data of overall (average over 50 d incubation) changes in the concentration of NH_4_^+^-N (mg kg^-1^) in soil amended with urea N with or without nitrification inhibitor. (DOCX)

S2 Table Data of overall (average over 50 d incubation) changes in the concentration of NO_2_^-^-N (mg kg^-1^) in soil amended with urea N with or without nitrification inhibitor. (DOCX)

S3 Table Data of overall (average over 50 d incubation) changes in the concentration of NO_3_^-^-N (mg kg^-1^) in soil amended with urea N with or without nitrification inhibitor. (DOCX)

S4 Table Data of dynamic changes in the concentration of total mineral N (mg kg^-1^) in soil amended with urea N with or without nitrification inhibitor. (DOCX)
