# Supplementary material for: Dicyandiamide has more inhibitory activities on nitrification than thiosulfate
Source: PLoS One. 2018 Aug 14;13(8):e0200598. doi: 10.1371/journal.pone.0200598 (PMC6091914; doi:10.1371/journal.pone.0200598)
Supplement: S1 Table — (DOCX) [file pone.0200598.s001.docx]

| Treatments | NH_4_^+^-N (mg kg^-1^) | standard deviations of NH_4_^+^-N |
| --- | --- | --- |
| CK | 10.1 | 0.97 |
| N | 74.49 | 1.34 |
| N+DCD | 200.51 | 3.45 |
| N+K_2_S_2_O_3_ | 101.7 | 1.53 |

**S1: Data of Fig 2 Overall ( average over 50 d incubation ) changes in the concentration of NH_4_^+^-N (mg kg^-1^) in soil amended with urea N with or without nitrification inhibitor**
