# Supplementary material for: Dicyandiamide has more inhibitory activities on nitrification than thiosulfate
Source: PLoS One. 2018 Aug 14;13(8):e0200598. doi: 10.1371/journal.pone.0200598 (PMC6091914; doi:10.1371/journal.pone.0200598)
Supplement: S2 Table — (DOCX) [file pone.0200598.s002.docx]

**S2: Data of overall (average over 50 d incubation) changes in the concentration of NO_2_^-^-N (mg kg^-1^) in soil amended with urea N with or without nitrification inhibitor**

| Treatments | NO_2_^-^-N (mg kg^-1^) | standard deviations of NO_2_^-^-N |
| --- | --- | --- |
| CK | 0.71 | 0.058 |
| N | 6.1 | 0.2 |
| N+DCD | 0.184 | 0.031 |
| N+K_2_S_2_O_3_ | 6.9 | 0.261 |
