# Supplementary material for: Dicyandiamide has more inhibitory activities on nitrification than thiosulfate
Source: PLoS One. 2018 Aug 14;13(8):e0200598. doi: 10.1371/journal.pone.0200598 (PMC6091914; doi:10.1371/journal.pone.0200598)
Supplement: S3 Table — (DOCX) [file pone.0200598.s003.docx]

| Treatments | NO_3_^-^-N (mg kg^-1^) | standard deviations of NO_3_^-^-N |
| --- | --- | --- |
| CK | 103.23 | 4.9 |
| N | 164.77 | 3.97 |
| N+DCD | 120.11 | 3.44 |
| N+K_2_S_2_O_3_ | 144.41 | 2.6 |

**S3: Data of overall (average over 50 d incubation) changes in the concentration of NO_3_^-^-N (mg kg^-1^) in soil amended with urea N with or without nitrification inhibitor**
