# Supplementary material for: Dicyandiamide has more inhibitory activities on nitrification than thiosulfate
Source: PLoS One. 2018 Aug 14;13(8):e0200598. doi: 10.1371/journal.pone.0200598 (PMC6091914; doi:10.1371/journal.pone.0200598)
Supplement: S4 Table — (DOCX) [file pone.0200598.s004.docx]

**S4: Data of dynamic changes in the concentration of total mineral N (mg kg^-1^) in soil amended with urea N with or without nitrification inhibitor**

| Incubation days | CK | standard deviations of CK | N | standard deviations of N | N+DCD | standard deviations of N+DCD | N+K_2_S_2_O_3_ | standard deviations of N+K_2_S_2_O_3_ |
| --- | --- | --- | --- | --- | --- | --- | --- | --- |
| 1 | 100.1 | 2.16 | 298.95 | 9.63 | 300.59 | 2.57 | 222.65 | 2.21 |
| 5 | 97.64 | 5.74 | 319.33 | 9.41 | 320.15 | 5.87 | 304.6 | 4.36 |
| 10 | 122.93 | 7.1 | 330.4 | 2.11 | 348.5 | 8.87 | 337.27 | 8.29 |
| 15 | 99.74 | 3.73 | 212.11 | 10.26 | 329.61 | 4.61 | 287.62 | 7.36 |
| 20 | 112.91 | 5.93 | 210.55 | 4.8 | 345.63 | 6.03 | 250.33 | 12.5 |
| 30 | 126.88 | 2.89 | 196.8 | 27.12 | 352.61 | 6.5 | 215.49 | 4.32 |
| 40 | 125.93 | 19.38 | 222.89 | 14.63 | 325.12 | 24.55 | 214.11 | 12.22 |
| 50 | 126.21 | 4.97 | 179.88 | 14.41 | 233.46 | 1.36 | 189.88 | 11.2 |
